# Walking Speed and Brain Glucose Uptake are Uncoupled in Patients with Multiple Sclerosis

**DOI:** 10.3389/fnhum.2015.00084

**Published:** 2015-02-18

**Authors:** John H. Kindred, Jetro J. Tuulari, Marco Bucci, Kari K. Kalliokoski, Thorsten Rudroff

**Affiliations:** ^1^Department of Health and Exercise Science, Colorado State University, Fort Collins, CO, USA; ^2^Turku PET Centre, Turku University Hospital, University of Turku, Turku, Finland

**Keywords:** positron emission tomography, glucose uptake, multiple sclerosis, walking, brain activity, movement disorder

## Abstract

Motor impairments of the upper and lower extremities are common symptoms of multiple sclerosis (MS). While some peripheral effects like muscle weakness and loss of balance have been shown to influence these symptoms, central nervous system activity has not been fully elucidated. The purpose of this study was to determine if alterations in glucose uptake were associated with motor impairments in patients with multiple sclerosis. Eight patients with multiple sclerosis (four men) and eight sex matched healthy controls performed 15 min of treadmill walking at a self-selected pace, during which ≈322 MBq of the positron emission tomography (PET) glucose analog [^18^F]-fluorodeoxyglucose (FDG) was injected. Immediately after the cessation of walking, participants underwent PET imaging. Patients with MS had lower FDG uptake in ≈40% of the brain compared to the healthy controls (*p*_FWE-corr_ < 0.001, *q*_FDR-corr_ < 0.001, *k*_e_ = 93851) and walked at a slower speed [MS, 1.1 (0.2), controls 1.4 (0.1), m/s, *P* = 0.014]. Within the area of lower FDG uptake 15 regions were identified. Of these 15 regions, 13 were found to have strong to moderate correlations to walking speed within the healthy controls (*r* > −0.75, *P* < 0.032). Within patients with MS only 3 of the 15 regions showed significant correlations: insula (*r* = −0.74, *P* = 0.036), hippocampus (*r* = −0.72, *P* = 0.045), and calcarine sulcus (*r* = −0.77, *P* = 0.026). This data suggest that walking impairments in patients with MS may be due to network wide alterations in glucose metabolism. Understanding how brain activity and metabolism are altered in patients with MS may allow for better measures of disability and disease status within this clinical population.

## Introduction

Motor impairments of the upper and lower extremities are some of the most common symptoms in patients with MS (Fox et al., 2006). Previous investigations have shown muscle weakness, spasticity, and loss of coordination/balance as contributors to these motor decrements (Rizzo et al., 2004; Fritz et al., 2014; Wagner et al., 2014). One area that has been less studied is how alterations in motor patterns generated within the central nervous system (CNS) may play a role. The most common methods for elucidating information about CNS activity during motor task performance are functional magnetic resonance imaging (fMRI) and electroencephalography (EEG). A major limitation of fMRI is that brain activity can only be measured while an individual is positioned within the MR camera (Gramann et al., 2014). While EEG is able to measure activity during walking, it can be hampered by interference and is unable to measure subcortical areas (Filippi et al., 2002).

An alternative to fMRI and EEG is positron emission tomography (PET). Using the PET glucose analog [^18^F]-fluorodeoxyglucose (FDG), the brains utilization/uptake of FDG can be quantified. Glucose is one of the main substrates the brain uses to generate ATP. By measuring FDG uptake into the CNS estimates of brain activity can be made (Ginsberg et al., 1988; Niccolini et al., 2015). FDG PET also allows for the quantification of all brain regions during any type of free living activity, such as walking, running, or driving a car (Tashiro et al., 2001; Jeong et al., 2006; La Fougere et al., 2010).

Utilizing FDG PET at rest, Roelcke et al. (1997) and Bakshi et al. (1998) found reduced glucose metabolism within the brain of patients with MS compared to healthy controls. Bakshi et al. (1998) also suggested that cerebral dysfunction and neuronal system disconnection, or uncoupling, may play an important role in the symptoms of MS. The purpose of this study was to determine the associations between brain activity, as measured by FDG uptake, and walking ability in patients with MS and healthy controls. We hypothesized that patients with MS would have lower FDG uptake during walking compared to controls, and that associations with brain regions responsible for motor task performance/control are altered in patients with MS.

## Materials and Methods

Basic descriptions of the methods utilized in this investigation are provided here. A more detailed explanation can be found in Rudroff et al. (2014). All testing was performed between the hours of 0700 and 1100 to reduce the influence of fatigue on patients with MS.

### Participant recruitment

**Figure 1:**
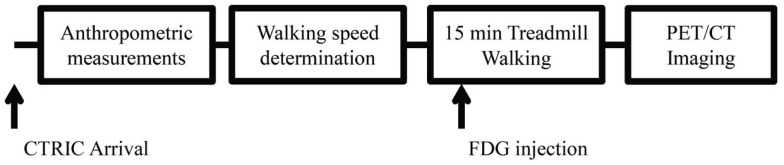
**Representative image of the experimental timeline**.

Eight patients with MS and eight controls participated in this study. Basic inclusion criteria for patients with MS included positive MS diagnosis, ability to walk 15 min without assistance, and no change in disease status/had a relapse within the previous 3 months. Controls were sex matched and without known cardiovascular, neurological, or musculoskeletal disease. All procedures were approved by the Colorado Multi Institutional Review Board and all experiments conformed to the Declaration of Helsinki. Upon arrival to the Colorado Translational Research Imaging Center all participants signed informed consent. Measurements for height, weight, and age were obtained for all participants. Patients with MS were assessed for disability utilizing the patient determined disease steps (PDDS) and the modified Ashworth scale for grading spasticity (MASS). Figure 1 is a representation of the experimental timeline. Participant characteristics are displayed in Table 1.

**Table 1:** **Subject characteristics and clinical measures**.

*N*	MS	CON	*P*-value
	
	8 (4 women)	8 (4 women)	
Age (years)	44.9 (8.6)	37.9 (8.4)	0.122
Height (cm)	175 (8)	176 (7)	0.949
Weight (kg)	78.2 (3.3)	78.2 (6.3)	0.982
Disease duration (years)	8.9 (6.2)		
PDDS	2 (0-4)		
MASS	1 (0-1.5)		
Walking speed (m/s)*	1.1 (0.2)	1.4 (0.1)	0.014
RPE	1.7 (1.4)	1.5 (1.1)	0.681

*Age, height, weight, disease duration, walking speed, and RPE are reported as mean (SD)*.

*PDDS, patients determined disease steps; and MASS, modified Ashworth scale for grading spasticity, are reported as median (range)*.

*P < 0.050.

### Walking test

Participants were asked to walk down a 60 m hallway 3–5 times. The time it took them to walk the middle 20 m was timed with a handheld stopwatch. The two closest times were averaged and this speed was set as the initial speed of the treadmill. After their comfortable walking speed was calculated and set on the treadmill, participants began 15 min of treadmill walking. Any adjustments to this speed were made within the first 2 min. Two minutes after the start of treadmill walking ≈321.9 MBq of FDG was injected into an antecubital vein via a previously inserted catheter. During treadmill walking, participants were asked their rating of perceived exertion (RPE), measured on the 10 point Borg scale, every minute. At the conclusion of treadmill walking, participants were escorted to the PET/computed tomography (CT) camera, and within 2 min underwent the start of PET/CT imaging.

### PET/CT imaging

Positron emission tomography/CT imaging was performed on a Phillips Hybrid Gemini TF 64 camera (Philips Healthcare, Cleveland, OH, USA). PET images were acquired in list-mode and in 3-D mode, utilizing time-of-flight technology in order to improve the image contrast vs. noise. A standard Colorado Translational Research Imaging Center testing protocol was utilized. PET/CT images were acquired consecutively with the participants’ body secured to maintain co-registration of the images.

### Image analysis

Positron emission tomography images were cropped using Analyze 11.0 (Mayo Clinic, Rochester, MN, USA) to allow for analysis of the brain via the Statistical Parametric Mapping 8 (SPM8)^1^ toolbox for Matlab 2011a (The MathWorks, Inc., Natick, MA, USA). FDG PET images were then transformed into SUV parametric images using voxel by voxel calculation via the formula: SUV = Activity (kBq/cc)/((Injected Activity (MBq)/Body Weight (kg)). After SUV calculation, images were spatially normalized to a tracer specific template into Montreal Neurological Institute (MNI) space, as described in Tuulari et al. (2013). Images were smoothed at 10-mm Full Width at Half Maximum. Smoothed spatially normalized SUV images were then analyzed with SPM8.

A two-sample *t*-test batch process was performed within SPM8 to identify clusters of differing FDG uptake between the two groups (group 1 MS, group 2 CON), utilizing walking speed as a covariate, and a relative threshold masking set at 0.8. *T*-contrasts of “−1 1” and “1 −1” were tested with a *P-value* set to 0.01 and an extent threshold (*k*_e_) = to 0 (voxels). Whole brain and current cluster values were identified at the cluster level. The SUV for regions within the significant cluster level were determined using the automatic anatomic labeling (AAL) template extracted with the marsbar^2^ SPM toolbox. Visual inspection of the overlap between ROIs and SPM threshold output overlaid on the AAL template was performed using MRIcron (Rorden and Brett, 2000).

### Statistical analysis

Whole brain statistical analysis was performed within SPM8 toolbox and ROI-based Pearson’s correlations, brain region to walking speed, were performed with SPSS 22 (IBM Corp, Armonk, NY, USA). Participant characteristics were compared utilizing unpaired *t*-tests. For analysis performed in SPSS, a significant α was set at <0.050.

## Results

### Subject characteristics

There were no differences between the MS and CON group for age, height, or weight (*P* > 0.122). Patients with MS were classified as having mild disability determined from scores of the MASS and PDDS. Patients with MS walked at a slower self-selected speed than controls (*P* = 0.014), but without a difference in RPE (*P* = 0.681). All participant characteristics are provided in Table 1. These characteristics have been previously reported (Rudroff et al., 2014).

### SPM analysis

**Figure 2:**
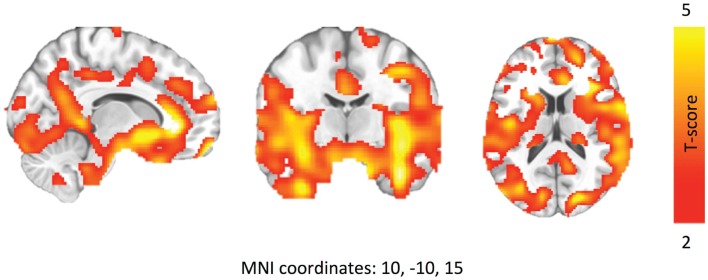
**Brain regions where patients with MS have lower FDG uptake after walking challenge**. Walking speed has been modeled out as a nuisance factor. Data are thresholded at *P* < 0.01, FDR corrected.

**Figure 3:**
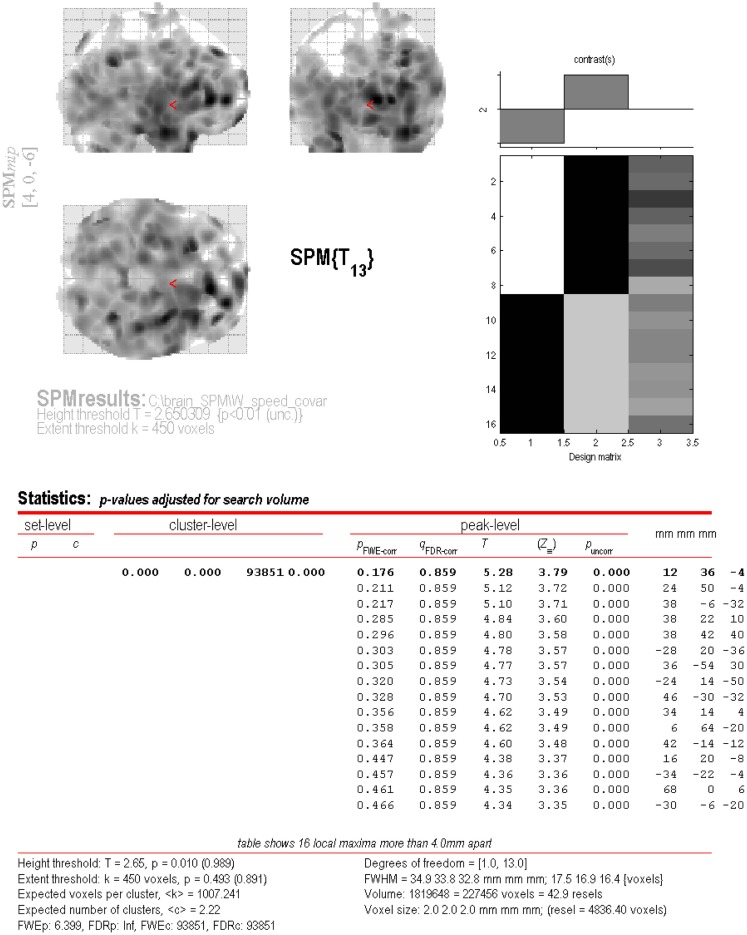
**SPM output of the areas of lower FDG uptake in patients with MS compared to controls**. Within cluster information is displayed for the large cluster (*p*_FWE-corr_ < 0.001, *q*_FDR-corr_ < 0.001, *k*_e_ = 93851) identified during analysis.

[^18^F]-fluorodeoxyglucose uptake in patients with MS was lower compared to controls, represented by one large cluster (*p*_FWE-corr_ < 0.001, *q*_FDR-corr_ < 0.001, *k*_e_ = 93851) (Figure 2). This cluster represented ~40% of total brain volume (227456 voxels). No cluster or peak-level regions were found to have higher FDG uptake in patients with MS. Figure 3 is the cluster-level SPM output for the identified cluster, displaying peak-level information. Table 2 displays SPM output with associated AAL labels defined using MRIcron.

**Table 2:** **SPM analysis of cluster-level differences between patients with MS and healthy controls**.

*T*	*Z*	*p*_uncorr_	MNI coordinates			Region
5.28	3.79	0.000	12	36	−4	Cingulum_Ant_R^a^
5.12	3.72	0.000	24	50	−4	Frontal_Sup_Orb_R^a^
5.10	3.71	0.000	38	−6	−32	Fusiform_R
4.84	3.60	0.000	38	22	10	Frontal_Inf_Tri_R
4.80	3.58	0.000	38	42	40	Frontal_Mid_R
4.78	3.57	0.000	−28	20	−36	Temporal_Pole_Mid_L
4.77	3.57	0.000	36	−54	30	Angular_R^a^
4.73	3.54	0.000	−24	14	−50	Fusiform_L^a^
4.70	3.53	0.000	46	−30	−32	Cerebelum_6_R^a^
4.62	3.49	0.000	34	14	4	Insula_R
4.62	3.49	0.000	6	64	−20	Frontal_Sup_Orb_R
4.60	3.48	0.000	42	−14	−12	Hippocampus_R^a^
4.38	3.37	0.000	16	20	−8	Cuadate_R
4.36	3.36	0.000	−34	−22	−4	Hippocampus_L^a^
4.35	3.36	0.000	68	0	6	Temporal_Sup_R^a^
4.34	3.35	0.000	−30	−6	−20	Hippocampus_L

*^a^Visually placed in nearest labeled area within the automatic anatomical labeling (AAL) MRIcron template*.

*All locations represent areas of lower activity in the MS group vs. the CON group*.

### ROI and walking speed correlations

**Figure 4:**
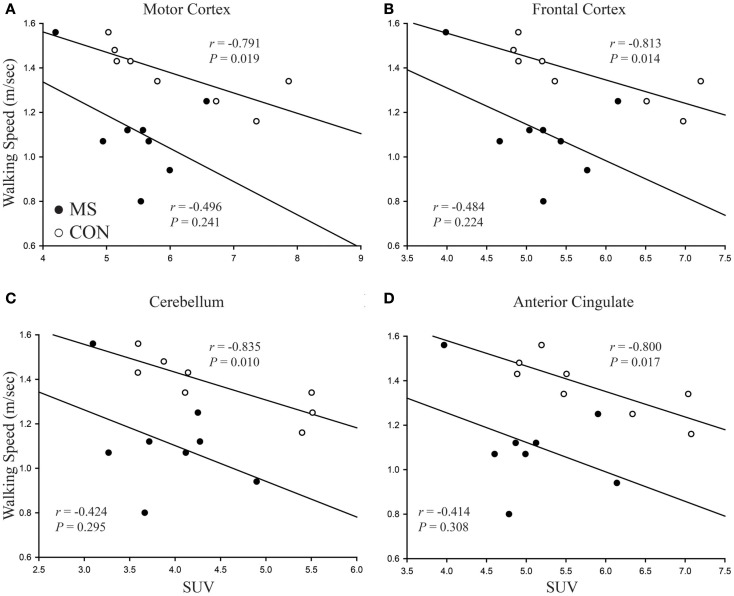
**Visual representation of correlations between walking speed and brain region FDG uptake**. In each case, the strength of the correlation is less in patients with MS compared to controls. As well as correlations being weaker, patients with MS show no statistical significance while correlations for the control group all reach statistical significance. **(A)** Motor cortex, **(B)** frontal cortex, **(C)** cerebellum, **(D)** anterior cingulate.

Strong to moderate Pearson correlations were found in 13 out of 15 regions identified from SPM analysis to walking speed within the control group (*r* > −0.75, *P* < 0.032). Within the MS group only three regions, the insula (*r* = −0.74, *P* = 0.036), hippocampus (*r* = −0.72, *P* = 0.045), and calcarine sulcus (*r* = −0.77, *P* = 0.026) were found to have statistically significant correlations. In both groups neither the thalamus nor caudate had a significant correlation, although within the control group it was borderline (0.051 ≤ *P* < 0.063). In general all other areas, while not reaching statistical significance, had a lower Pearson *r*-value compared to the same region within the control group. The uncoupling of FDG uptake and walking speed is visualized for four regions in Figure 4. Table 3 lists the all *r*-values and associated *P*-values.

**Table 3:** **Pearsons’ correlations between walking speed and brain region FDG uptake**.

Brain region	MS		CON	
	*r*-value	*P*-value	*r*-value	*P*-value
Frontal cortex	−0.484	0.224	−0.813	0.014*
Occipital cortex	−0.664	0.073	−0.786	0.021*
Lateral temporal cortex	−0.664	0.073	−0.818	0.013*
Medial temporal cortex	−0.636	0.090	−0.832	0.010*
Motor cortex	−0.496	0.241	−0.791	0.019*
Cerebellum	−0.424	0.295	−0.835	0.010*
Insula	−0.740	0.036*	−0.817	0.013*
Hippocampus	−0.718	0.045*	−0.751	0.032*
Anterior cingulum	−0.414	0.308	−0.800	0.017*
Precuneus	−0.603	0.113	−0.799	0.017*
Calcarine	−0.767	0.026*	−0.750	0.032*
Lingual	−0.680	0.064	−0.850	0.007*
Fusiform	−0.626	0.097	−0.851	0.007*
Thalamus	−0.324	0.433	−0.680	0.063
Caudate	−0.557	0.151	−0.704	0.051

*P < 0.050.

## Discussion

Results from SPM analysis showed that patients with MS had lower FDG uptake in ~40% of the brain compared to controls as well as weaker associations with preferred walking speed. Interestingly, the motor cortex, the origin of central motor command, was not found to be associated to walking speed in patients with MS (*r* = −0.496, *P* = 0.241). This data suggest that alterations in task performance in patients with MS, such as walking, may be due to network wide uncoupling of CNS activity.

### Lower FDG uptake

Roelcke et al. (1997) previously showed a reduced central metabolic rate of glucose in patients with MS. Possible explanations are reduced brain volume and enlarged ventricles (Grassiot et al., 2009), which are common effects of MS, and altered glucose metabolism that has been shown in MS and other neurological diseases (Mathur et al., 2014). One hypothesis that encompasses both of these factors is that mitochondrial dysfunction can lead to neurodegeneration (Su et al., 2009, 2013; Cambron et al., 2012). Amorini et al. (2014) reported greatly elevated serum lactate levels in patients with MS. They suggested this is due to mitochondrial dysfunction, which results in a reduced oxidative capacity, and in turn to a higher lactate concentration. Smith et al. (2003) investigated the effect of infused lactate on resting CNS FDG uptake and found that FDG uptake was reduced with lactate infusion. During exercise, it has also been shown that brain FDG uptake can be reduced. This reduction was correlated with the increase in lactate production (Kemppainen et al., 2005), although the intensities at which this was found to happen were 30, 50, and 75% ${\dot{\text{V}}\text{O}}_{2\max}$. Even though these patients with MS were only mildly disabled, a reduced brain volume and increased lactate utilization could be driving the lower FDG uptake observed in this study.

### Reduced associations between brain FDG uptake and walking speed

Much of our knowledge of brain function has been obtained from lesion studies. In these studies, focal lesions are created or patients with naturally occurring lesions were studied. A hallmark of MS is demyelinated lesions, which can be either active or dormant, throughout the CNS (Kutzelnigg and Lassmann, 2014). Often time’s clinical disability can be linked to the location and activity of these lesions (Rocca et al., 2002; Gil Moreno et al., 2013). While no MRI measurements were performed for this study, it is unlikely that any lesions the patients with MS had were all in the same locations.

In the control group, we found moderate to strong correlations with walking speed for most of the brain regions within the identified cluster. Many of these regions are involved in visual–spatial processing, sensory/motor integration, and executive function. It has been reported that the neural network for motor task performance is highly integrative, and not limited to areas like the motor cortex and supplemental motor areas, and can change depending on the task being performed (Neely et al., 2013). These areas of cortical activity have also been identified during walking and running in other studies using near infrared spectroscopy (NIRS) (Suzuki et al., 2004; Koenraadt et al., 2014) as well as during imagined walking with fMRI (Bakker et al., 2008). In patients with MS, however, it appears that these network connections are altered, suggesting a decoupling effect of brain activity and motor performance. Only the insular cortex, calcarine sulcus, and hippocampus had a significant association with walking speed in patients with MS. The strength of these correlations was also very similar to those within the healthy control group. Interestingly, the motor cortex in the control group showed a strong correlation to walking speed (*r* = −0.791, *P* = 0.019). The contributions of the motor cortex to steady state walking is not completely understood, with conflicting reports of its activity and importance being stated by multiple sources (Bakker et al., 2008; La Fougere et al., 2010; Petersen et al., 2012; Koenraadt et al., 2014). During gait challenges, it has also been shown that areas like the supplemental motor areas and prefrontal areas are more active to account for the continuous alterations necessary to navigate the challenges (Suzuki et al., 2004; Bakker et al., 2008; Koenraadt et al., 2014). With the increase in disability and disease progression, it is possible that these areas are used to a greater extend to maintain ambulation in patients with MS and the inability to fully utilize them during walk may contribute to the slower walking speed observed in these patients.

The calcarine sulcus is located within the primary visual cortex, within the occipital lobe. Visual feedback is important for most motor tasks (Zhang et al., 2011; Sarlegna and Mutha, 2014). It allows for the proper interpretation of the body in the environment. Visual feedback also plays an important role in the maintenance of balance (Prosperini et al., 2010), which is often impaired in patients with MS. The insular cortex is a located medial to the temporal lobe and is known as a motor/sensory association area. The integration of sensory and motor cues are necessary for the continuous updates of motor patterns (Smucny et al., 2013), ensuring efficient task performance. The hippocampus connects to the medial temporal lobe. This area has been implicated in motor task performance through fMRI studies of recalled walking (La Fougere et al., 2010; Wutte et al., 2012). It is believed that this area stores the motor patterns that are recalled during walking. Recall of these motor patterns would mostly occur through connections with the hippocampus as well as sensory/motor connections throughout the cortex of the frontal, parietal, and temporal lobes.

### Potential physiological mechanisms for walking impairments in patients with MS

Demyelinating lesions often occur throughout the CNS, with no two individuals having lesions at the exact same loci. The decoupling of the CNS and motor task performance may partially explain why many symptoms, such as difficulties walking, are shared between so many patients with MS. To maintain mental and physical function the brain forms new connections within itself to compensate for damage. This plasticity may result in the network wide alterations in glucose uptake, which we show is uncoupled with motor task performance. It is unclear whether these alterations in glucose uptake are causative of disability, or compensatory to maintain function. Further research is necessary to elucidate how alterations in CNS activity influence motor task performance in patients with MS.

### Methodological considerations

One limitation of this investigation is the lack of MRI data. Combining structural information like brain volume as well as lesion locations could provide additional insight in to why certain areas were correlated with walking speed while others were not. Brain atrophy is very common in patients with MS. The normalization of their PET image to a standard template could introduce error, which increases with greater atrophy. As the amount of atrophy is increased an SUV image would be stretched more to fit the standard template. If varying amounts of atrophy within the MS existed, it could in part explain the lack of correlation between brain ROI and walking speed. The average disease duration of patients with MS in this study was 8.9 years, with a range of 1–19 years, so varying amounts of atrophy can be expected. However, the three ROIs that were found to be significantly correlated with walking speed in the MS group, has similar *r*-values and *P*-values as that of the control group. Since atrophy is common in MS using individualized MR images for normalization may be able to account for the variance due to atrophy and should be performed in future studies. Another limitation is the lack of a baseline FDG PET image so that relative activation/deactivation could be estimated for the groups. Future studies utilizing both MRI and PET may provide greater information on the associations between structure and function within the human brain. Another aspect to consider is the importance of spinal cord activated motor commands from central pattern generators. It is possible that an increased reliance on these motor neurons could reduce correlations with walking performance and the brain.

## Conclusion

Mildly disabled patients with MS have been shown to decrements in function task performance. In this sample, these decrements were reflected by a significantly slower self-selected walking speed. These patients also demonstrated reduced FDG uptake into ~40% of the brain. Only 3 out of 15 regions identified within the patients with MS, compared to 13 out of 15 regions in healthy controls, were found to be correlated with their walking speed. This may suggest a decoupling of brain glucose utilization and motor task performance. Whether this decoupling is a compensatory mechanism to maintain function or contributes to the decrements in motor task performance requires further studies. Future research studies need to be conducted to identify how to preserve the associations between brain glucose uptake and motor task performance in order to lessen the effects motor decrements have on the functional abilities of patients with MS.

## Author Contributions

JK contributed to (1) conception and design of the experiments; (2) collection, analysis, and interpretation of data; and (3) drafting the article and revising it critically for important intellectual content. JT and MB contributed to (1) analysis and interpretation of data; and (2) preparation of figures and tables. KK contributed to (1) interpretation of data; and (2) drafting the article and revising it critically for important intellectual content. TR contributed to (1) conception and design of the experiments; (2) collection, analysis, and interpretation of data; (3) analysis and interpretation of data; and (4) drafting the article and revising it critically for important intellectual content. All authors approved the final version of the manuscript.

## Conflict of Interest Statement

The authors declare that the research was conducted in the absence of any commercial or financial relationships that could be construed as a potential conflict of interest.

## Footnotes

^1^http://www.fil.ion.ucl.ac.uk/spm/

^2^http://marsbar.sourceforge.net

## References

[B1] Amorini A. M. Nociti V. Petzold A. Gasperini C. Quartuccio E. Lazzarino G. (2014). Serum lactate as a novel potential biomarker in multiple sclerosis. Biochim. Biophys. Acta 1842, 1137–1143. 10.1016/j.bbadis.2014.04.005 24726946

[B2] Bakker M. De Lange F. P. Helmich R. C. Scheeringa R. Bloem B. R. Toni I. (2008). Cerebral correlates of motor imagery of normal and precision gait. Neuroimage 41, 998–1010. 10.1016/j.neuroimage.2008.03.020 18455930

[B3] Bakshi R. Miletich R. S. Kinkel P. R. Emmet M. L. Kinkel W. R. (1998). High-resolution fluorodeoxyglucose positron emission tomography shows both global and regional cerebral hypometabolism in multiple sclerosis. J. Neuroimaging 8, 228–234. 9780855 10.1111/jon199884228

[B4] Cambron M. D’Haeseleer M. Laureys G. Clinckers R. Debruyne J. De Keyser J. (2012). White-matter astrocytes, axonal energy metabolism, and axonal degeneration in multiple sclerosis. J. Cereb. Blood Flow Metab. 32, 413–424. 10.1038/jcbfm.2011.193 22214904 PMC3293127

[B5] Filippi M. Rocca M. A. Colombo B. Falini A. Codella M. Scotti G. (2002). Functional magnetic resonance imaging correlates of fatigue in multiple sclerosis. Neuroimage 15, 559–567. 10.1006/nimg.2001.1011 11848698

[B6] Fox R. J. Bethoux F. Goldman M. D. Cohen J. A. (2006). Multiple sclerosis: advances in understanding, diagnosing, and treating the underlying disease. Cleve. Clin. J. Med. 73, 91–102. 10.3949/ccjm.73.1.91 16444920

[B7] Fritz N. E. Marasigan R. E. Calabresi P. A. Newsome S. D. Zackowski K. M. (2014). The impact of dynamic balance measures on walking performance in multiple sclerosis. Neurorehabil. Neural Repair 29, 62–69. 10.1177/1545968314532835 24795162 PMC4216642

[B8] Gil Moreno M. J. Cerezo Garcia M. Marasescu R. Pinel Gonzalez A. Lopez Alvarez L. Aladro Benito Y. (2013). Neuropsychological syndromes in multiple sclerosis. Psicothema 25, 452–460. 10.7334/psicothema2012.308 24124777

[B9] Ginsberg M. D. Chang J. Y. Kelly R. E. Yoshii F. Barker W. W. Ingenito G. (1988). Increases in both cerebral glucose untilization and blood flow during execution of a somatosensory task. Ann. Neurol. 23, 9. 10.1002/ana.410230208 3259852

[B10] Gramann K. Ferris D. P. Gwin J. Makeig S. (2014). Imaging natural cognition in action. Int. J. Psychophysiol. 91, 22–29. 10.1016/j.ijpsycho.2013.09.003 24076470 PMC3983402

[B11] Grassiot B. Despranges B. Eustache F. Defer G. (2009). Quantification and clinical relevance of brain atrophy in multiple sclerosis: a review. J. Neurol. 256, 1397–1412 10.1007/s00415-009-5108-4 19353226

[B12] Jeong M. Tashiro M. Singh L. N. Yamaguchi K. Horikawa E. Miyake M. (2006). Functional brain mapping of actual car-driving using [^18^F]FDG-PET. Ann. Nucl. Med. 20, 6. 10.1007/BF02984660 17294673

[B13] Kemppainen J. Aalto S. Fujimoto T. Kalliokoski K. K. Langsjo J. Oikonen V. (2005). High intensity exercise decreases global brain glucose uptake in humans. J. Physiol. 568, 323–332. 10.1113/jphysiol.2005.091355 16037089 PMC1474763

[B14] Koenraadt K. L. Roelofsen E. G. Duysens J. Keijsers N. L. (2014). Cortical control of normal gait and precision stepping: an fNIRS study. Neuroimage 85(Pt 1), 415–422. 10.1016/j.neuroimage.2013.04.070 23631980

[B15] Kutzelnigg A. Lassmann H. (2014). Pathology of multiple sclerosis and related inflammatory demyelinating diseases. Handb. Clin. Neurol. 122, 15–58. 10.1016/b978-0-444-52001-2.00002-9 24507512

[B16] La Fougere C. Zwergal A. Rominger A. Forster S. Fesl G. Dieterich M. (2010). Real versus imagined locomotion: a [18F]-FDG PET-fMRI comparison. Neuroimage 50, 1589–1598. 10.1016/j.neuroimage.2009.12.060 20034578

[B17] Mathur D. López-Rodas G. Casanova B. Marti M. B. (2014). Perturbed glucose metabolism: insights into multiple sclerosis pathogenesis. Front Neurol. 5:250. 10.3389/fneur.2014.00250 25520698 PMC4249254

[B18] Neely K. A. Coombes S. A. Planetta P. J. Vaillancourt D. E. (2013). Segregated and overlapping neural circuits exist for the production of static and dynamic precision grip force. Hum. Brain Mapp. 34, 698–712. 10.1002/hbm.21467 22109998 PMC3292669

[B19] Niccolini F. Su P. Politis M. (2015). PET in multiple sclerosis. Clin. Nucl. Med. 40, e46–52 10.1097/RLU.0000000000000359 24561681

[B20] Petersen T. H. Willerslev-Olsen M. Conway B. A. Nielsen J. B. (2012). The motor cortex drives the muscles during walking in human subjects. J. Physiol. 590, 2443–2452. 10.1113/jphysiol.2012.227397 22393252 PMC3424763

[B21] Prosperini L. Leonardi L. De Carli P. Mannocchi M. L. Pozzilli C. (2010). Visuo-proprioceptive training reduces risk of falls in patients with multiple sclerosis. Mult. Scler. 16, 491–499. 10.1177/1352458509359923 20150396

[B22] Rizzo M. A. Hadjimichael O. C. Preiningerova J. Vollmer T. L. (2004). Prevalence and treatment of spasticity reported by multiple sclerosis patients. Mult. Scler. 10, 589–595 10.1191/1352458504ms1085oa 15471378

[B23] Rocca M. A. Falini A. Colombo B. Scotti G. Comi G. Filippi M. (2002). Adaptive functional changes in the cerebral cortex of patients with nondisabling multiple sclerosis correlate with the extent of brain structural damage. Ann. Neurol. 51, 330–339. 10.1002/ana.10120 11891828

[B24] Roelcke U. Kappos L. Lechner-Scott J. Brunnschweiler H. Huber S. Ammann W. (1997). Reduced glucose metabolism in the frontal cortex and basal ganglia of multiple sclerosis patients with fatigue: a ^18^F-fluorodeoxyglucose positron emission tomography study. Neurology 48, 6. 10.1212/WNL.48.6.1566 9191767

[B25] Rorden C. Brett M. (2000). Stereotaxic display of brain lesions. Behav. Neurol. 12, 191–200. 11568431 10.1155/2000/421719

[B26] Rudroff T. Kindred J. H. Koo P. J. Karki R. Hebert J. R. (2014). Asymmetric glucose uptake in leg muscles of patients with multiple sclerosis during walking detected by [18F]-FDG PET/CT. NeuroRehabilitation 35, 813–823. 10.3233/nre-141179 25323085

[B27] Sarlegna F. R. Mutha P. K. (2014). The influence of visual target information on the online control of movements. Vision Res. 10.1016/j.visres.2014.07.001 25038472

[B28] Smith D. Pernet A. Hallett W. A. Bingham E. Marsden P. K. Amiel S. A. (2003). Lactate: a preferred fuel for human brain metabolism in vivo. J. Cereb. Blood Flow Metab. 23, 658–664. 10.1097/01.WCB.0000063991.19746.11 12796713

[B29] Smucny J. Rojas D. C. Eichman L. C. Tregellas J. R. (2013). Neuronal effects of auditory distraction on visual attention. Brain Cogn. 81, 263–270. 10.1016/j.bandc.2012.11.008 23291265 PMC3596875

[B30] Su K. Bourdette D. Forte M. (2013). Mitochondrial dysfunction and neurodegeneration in multiple sclerosis. Front. Physiol. 4:169 10.3389/fphys.2013.00169 23898299 PMC3722885

[B31] Su K. G. Banker G. Bourdette D. Forte M. (2009). Axonal degeneration in multiple sclerosis: the mitochondrial hypothesis. Curr. Neurol. Neurosci. Rep. 9, 411–417. 10.1007/s11910-009-0060-3 19664372 PMC2839873

[B32] Suzuki M. Miyai I. Ono T. Oda I. Konishi I. Kochiyama T. (2004). Prefrontal and premotor cortices are involved in adapting walking and running speed on the treadmill: an optical imaging study. Neuroimage 23, 1020–1026. 10.1016/j.neuroimage.2004.07.002 15528102

[B33] Tashiro M. Itoh M. Fujimoto T. Fujiwara T. Ota H. Kubota K. (2001). ^18^F-FDG PET mapping of regional brain activity in runners. J. Sports Med. Phys. Fitness 41, 7. 11317143

[B34] Tuulari J. J. Karlsson H. K. Hirvonen J. Hannukainen J. C. Bucci M. Helmio M. H. (2013). Weight loss after bariatric surgery reverses insulin-induced increases in brain glucose metabolism of the morbidly obese. Diabetes 62, 5. 10.2337/db12-1460/-/DC1 23493575 PMC3717871

[B35] Wagner J. M. Kremer T. R. Van Dillen L. R. Naismith R. T. (2014). Plantarflexor weakness negatively impacts walking in persons with multiple sclerosis more than plantarflexor sapsticity. Arch. Phys. Med. Rehabil. 95, 1358–1365. 10.1016/j.apmr.2014.01.030 24582617 PMC4152915

[B36] Wutte M. G. Glasauer S. Jahn K. Flanagin V. L. (2012). Moving and being moved: differences in cerebral activation during recollection of whole-body motion. Behav. Brain Res. 227, 21–29. 10.1016/j.bbr.2011.09.042 22040905

[B37] Zhang H. Xu L. Wang S. Xie B. Guo J. Long Z. (2011). Behavioral improvements and brain functional alterations by motor imagery training. Brain Res. 1407, 38–46. 10.1016/j.brainres.2011.06.038 21764038

